# The SAFE strategy for trachoma control: poised for rapid scale-up

**Published:** 2014

**Authors:** Paul Emerson

**Affiliations:** Director: International Trachoma Initiative, Decatur, USA. Pemerson@trachoma.org

**Figure:**
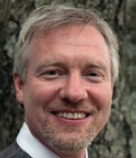
Paul Emerson

In 1998, all member states of the United Nations signed up for the Global Alliance for the Elimination of Blinding Trachoma by 2020 (GET 2020) through World Health Assembly resolution 51.1. This called on member states to complete the mapping of blinding trachoma in the remaining endemic areas and implement the SAFE strategy for trachoma control. Compiled data held by the International Trachoma Initiative (ITI) and the World Health Organization (WHO) indicated that, by 2010, as many as 1,090 districts in 36 of the estimated 57 trachoma endemic countries had been mapped. Moreover, the full SAFE strategy, including mass drug administration with oral azithromycin (Zithromax^®^) donated by Pfizer, was underway in 347 districts.

Global progress in trachoma mapping and implementation of the integrated SAFE strategy (see panel) was reviewed by the International Task Force for Disease Eradication (ITFDE) in 2010.^1^ The ITFDE concluded that the elimination of blinding trachoma was achievable and that significant progress had been made. However, it also noted that the pace and scale of interventions needed to be accelerated in order to reach these goals. Progress in the scale of implementation of the ‘A’ component of the SAFE strategy from 2010 to 2014 is shown in Figure 1. In 2014, 25 trachoma endemic countries have planned – and are adequately resourced – to implement the SAFE strategy in 541 districts, a 55% increase since 2010.

**Figure:**
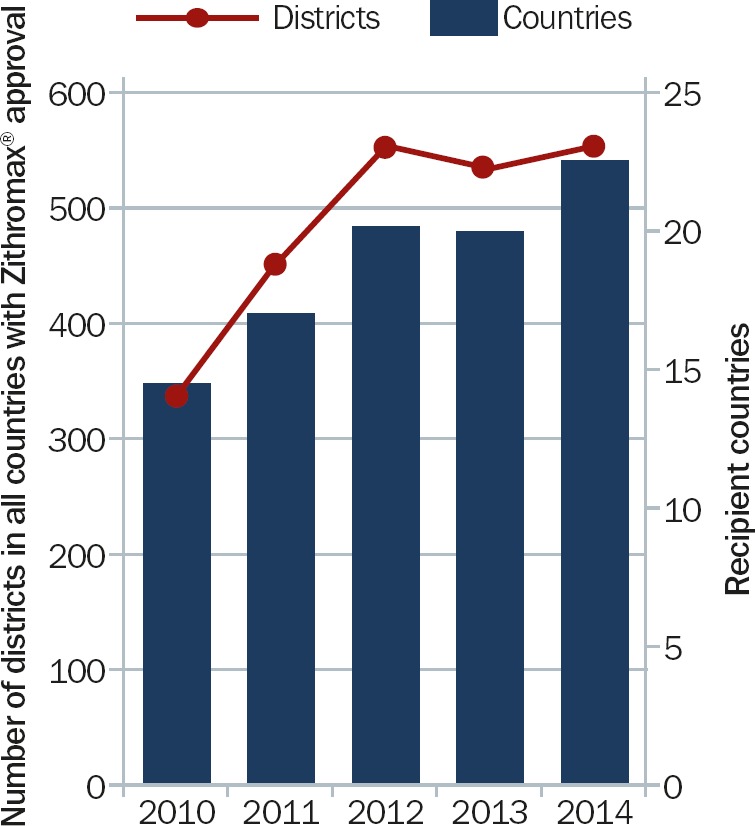
Figure 1. Expansion in countries and districts receiving Zithromax® for trachoma elimination 2010-2014

In 2012 the International Coalition for Trachoma Control (ICTC) produced a strategic plan that laid out actions to take, and milestones to meet, in order to achieve global elimination of blinding trachoma by 2020.^2^ This included a list of confirmed trachoma endemic countries, and another 1,293 districts suspected to be trachoma endemic, which needed to be mapped.

Sightsavers, in collaboration with ICTC partners, secured funding from the British government for the Global Trachoma Mapping Project (GTMP).^3^ Since the start of this project, 1,061 districts representing a population of 124 million people in 20 countries have been mapped^4^ and the GTMP has plans to survey an additional 862 districts (including districts in 11 additional countries).

The purpose of the GTMP is to provide the data required to chart the progress of the WHO-led GET 2020 Alliance, and to indicate which countries and districts require the greatest investment. Impact assessment data reported to ITI by the national programmes demonstrate that where trachoma is entrenched (baseline prevalence of TF in children aged 1–9 years is 30% and above), even 5 years of intervention with SAFE is insufficient to achieve the elimination goals of reducing TF to less than 5%, and of reducing the prevalence of TT in the whole population to less than 0.1%. These districts need to be immediately prioritised for urgent action and intensive intervention as we are working against the clock to reach elimination of blinding trachoma by 2020.

The ICTC membership have worked tirelessly with funding organisations, and, with generous support from the Queen Elizabeth Diamond Jubilee Trust, USAID, DFID, Lions Clubs International Foundation, the Conrad N Hilton Foundation and others, anticipate being able to bring in US $180 million in new funding for trachoma implementation over the next 5 years. Pfizer, which donates the antibiotic used in mass drug administration, is also preparing to donate over 100 million doses a year starting in 2015.

Rapid scale-up for maximum impact is ongoing, or planned, in Ethiopia, Kenya, Uganda, Senegal, Tanzania, Chad, Central African Republic, South Sudan, Nigeria, Malawi, Mozambique, Solomon Islands, Guinea, Yemen, and Zambia. Several other countries, such as Nepal, Sudan, Burkina Faso, Mali, Niger, Cameroon and Guinea-Bissau, are already implementing at or near national scale (where internal security allows it). With the confluence of data, financial resources, donated drugs, and – most importantly – political will to get the job done by the endemic countries, the GET 2020 Alliance is poised for the required scale-up, making the prospects for achieving the target of freeing the world of blinding trachoma that much more real.

**The SAFE strategy for trachoma control**

**Surgery:** For patients with severe blinding trachoma, eyelid surgery is needed to reposition turned-in eyelashes (trichiasis) so they do not scrape against the cornea.

**Antibiotics:** an annual dose of oral azithromycin or topical tetracycline is used to treat infection and decrease transmission in endemic districts.

**Facial cleanliness:** face washing helps to reduce transmission of trachoma by discouraging eye-seeking flies and washing away the bacteria they leave behind.

**Environmental improvements**, such as access to water and basic sanitation, reduce exposure and infection.

**What is ‘scale-up’?**

‘Scale-up’ means making a special effort to do something in a bigger way in order to improve some aspect of a population's health. Approaches to scaling up the SAFE strategy include improving the infrastructure for distribution of azithromycin, increasing the number of qualified surgeons who can provide trichiasis surgery, and enhancing the promotion of facial cleanliness, hygiene and environmental change in order to interrupt transmission.
